# Differences in hair coat condition between female and male mice occur through testicular-derived factors and are affected by the presence of another mouse

**DOI:** 10.1371/journal.pone.0324993

**Published:** 2025-06-18

**Authors:** Aisa Ozawa, Mei Imada, Ayane Hata, Kei Yokosuka, Motoharu Sakaue

**Affiliations:** Laboratory of Anatomy II, Department of Veterinary Medicine, School of Veterinary Medicine, Azabu University, Chuo-ku, Sagamihara, Kanagawa, Japan; University of Leipzig Faculty of Life Sciences: Universitat Leipzig Fakultat fur Lebenswissenschaften, GERMANY

## Abstract

An animal’s coat protects its body from ultraviolet rays, physical and chemical damage, and temperature changes. It also plays an important role in showing individual information like a pattern for the outside world. In addition, changes in the condition of an animal’s coat may occur due to health or psychological changes and are important indicators from the perspective of health management and the welfare of captive animals. Also, the quantity and texture of human hair play a role in the impression it gives to others; however, few reports have objectively quantified the appearance and detailed analysis of hair condition relative to the time of appearance. In this study, we quantified changes in hair coat condition and examined the underlying causes. The results indicated that changes in hair coat condition in male mice were significantly greater compared with that in female mice and were affected by testicular-derived factors and the presence or absence of cohabiting individuals. We also found that the condition of the coat is related to the length of the lift-edges cuticle region (LCR), which indicates that the cuticle condition affects the overall impression of the coat. This is the first report to show that changes in hair coat condition within sexual difference are related to the length of the LCR, suggesting that gender is a direct factor in the hair coat condition. Cohabitation, or the presence and relationship between one another, is an indirect factor.

## Introduction

The hair coat of animals plays a role in maintaining body temperature and protecting the skin from ultraviolet light or physical harm. Hair coat also provides information to its surroundings. For example, the hair coat shows a varying pattern between species and individuals and is associated with health conditions. Thus, the hair coat of animals provides a clue to an animal’s condition; however, the factors that directly affect hair coat condition are unclear.

During Japanese fancy mouse 1 (JF1/Ms) mouse breeding, we observed a difference between the hair coat conditions of males and females, in which the male mice exhibited an unkempt condition of the coat hair, whereas the female mice did not. Therefore, we hypothesized that sex differences may be attributed to the condition of the hair coat regardless health conditions. There are many reports implicating the sex hormones, androgen and estrogen. Androgens regulate hair follicle growth via androgen receptors on dermal papilla cells and exert different effects on hair follicles in different parts of the body. On the face, axilla, and chest, androgens promote hair growth, whereas on the scalp, they inhibit growth [1–3]. In addition, estrogen receptors are related to the hair cycle and are expressed differently in each follicle structure, dermal papilla cells, outer root sheath, inner root sheath, and bulge, in each hair cycle; estrogen administration delays hair growth and induces melanocyte activity [1,4–6]. Thus, sex hormones are a factor in hair loss and graying hair; however, a function of sex hormones as the factors that change the appearance of hair conditions remain unclear. The cuticle is the external layer of hair and protects it from environmental stimuli [7,8]. Loss or damage to the hair causes hair to split. The cuticle is composed of a multi-layer-like scale and the junction of the layers protects the inner hair from invasion by chemical substances or humidity. The loose junction readily invades and injures the inner hair. Thus, we examined the ratio of the lift-edges cuticle region (LCR) in full hair length relative to changes in hair condition. Few reports have shown a relationship between the lift-edges cuticle and hair coat condition; thus, we verified the degree of LCR that changed the hair condition. By clarifying the condition of the cuticle under changed hair conditions, the role of cuticle as the specific factors associated with the change in hair condition may be identified. Therefore, in this study, we examined the hair cuticle and the factors that alter the hair coat condition.

## Materials & methods

### Animals and treatment

The experimental protocols were approved by the Ethics Committee for Vertebrate Experiments at Azabu University (ID# 220210−8). JF1/Ms mice were obtained from National institute of genetics (Shizuoka, Japan) and, bred and propagated in a pathogen-free animal facility at Azabu University. Cohabitation started on 5-week-old mice and mice were randomized into the following groups: male single-breeding (n = 8), male pair-breeding (n = 20), female single-breeding (n = 9), female pair-breeding (n = 12), and female and male breeding (n = 4). A castration (n = 18) or sham operation (n = 16) was performed on 5-week-old mice under anesthesia (a combination anesthetic (0.3 mg/kg of medetomidine (DOMITOR, NIPPON ZENYAKU KOGYO, Fukushima, Japan), 4.0 mg/kg of midazolam (Fuji Pharma, Toyama, Japan), 5.0 mg/kg of butorphanol (Vetorphale, Meiji Animal Health Co., Tokyo, Japan). Euthanasia was performed by trained individual via cervical dislocation. The scoring hair coat condition was observed and the hairs of the thoracodorsal region were collected to determine the ratio of LCR as described below.

### The scoring hair coat condition and the ratio of LCR

The scoring hair coat condition was recorded every 2 weeks and the hairs for the measurement of the LCR were collected from the dorsal neck region of the mice. The hairs were covered in mounting medium (New M/X, MATSUNAMI GLASS, Osaka, Japan) for further observation. The length of the LCR and the full length of the hair shaft were assessed by light microscopy (Olympus fluorescent microscope DP70, Olympus, Tokyo, Japan). The ratio of LCR length versus full hair length was calculated and presented as the average of 10 hairs per mouse.

### Statistical analysis

All data are shown as the mean ± SE. Statistical analysis was performed using Bonferroni’s multiple comparisons test. The differences were considered to be statistically significant at P < 0.05 and P < 0.01 level and.

## Results

### Change of hair coat condition in JF1/Ms mice

**Fig 1:**
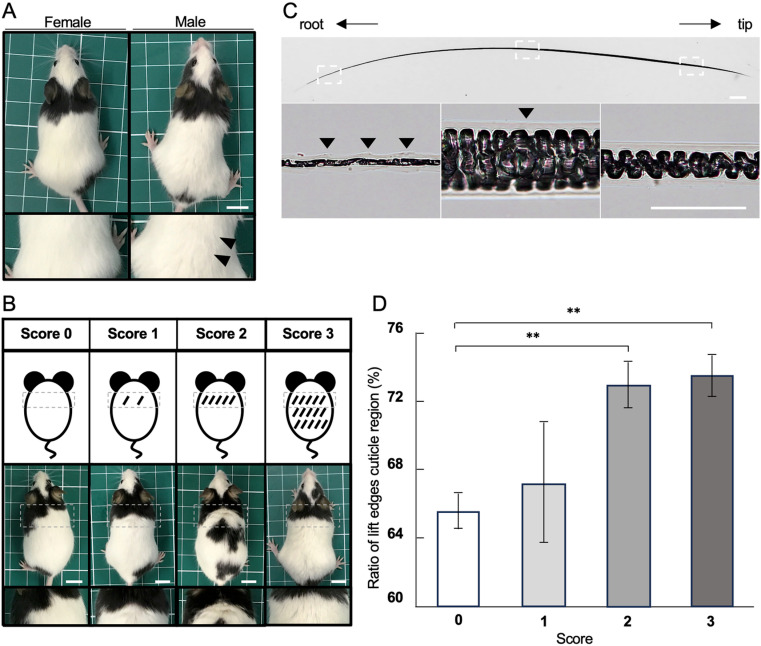
Scoring of hair coat conditions and the relation to the coat condition score and the ratio of LCR. (A) Hair coat condition in JF1/Ms mouse male and female 17-week-old mice. The male hair coat appeared rough (arrowhead). (B) The hair coat condition score. The squares with dotted lines in the upper and middle panels are images representing each score in the lower panels. The diagonal lines in upper panels indicate unkempt hair area. (C) Cuticle state analyzed by light microscopy. (D) The ratio of LCR for each hair coat condition score. The scale bar indicates 1 cm in A and B, 200 mm in upper panel C, and 50 mm in lower panel C. **P < 0.01.

Apparently, hair coat condition changes were observed in 17-week-old JF1/Ms mice (Fig 1A). The change was defined as hair standing on end and appearing unkempt, which was observed in male mice, but not in female mice. Thus, we examined the factors that cause hair condition changes in male mice. First, we defined a hair coat condition score to quantify the hairs of the dorsal neck (Fig 1B), because changes in the hair on the dorsal neck occur prior to that in the whole body. A score of 0 indicated no changes in the dorsal neck hair, a score of 1 indicated changes of less than 50% in the dorsal neck hair in sight, a score of 2 indicated overall changes in the dorsal neck hair, and a score of 3 indicated changes beyond the neck to the whole body (Fig 1B).

### Cuticle condition affects the hair coat condition score

The hair condition for each score was examined. The cuticle consists of overlapping-like scales at the outermost layer of the hair (Fig 1C). The LCR on the surface of the cuticle layer was observed from the hair root to approximately half of the length of the hair shaft (Fig 1C lower panels). After the LCR, the lift-edges cuticle was closed toward the hair tip. The cuticle area appeared smooth under an optical microscope (Fig 1C lower right panel). Various substances that can readily damage hair enter from the lift-edges cuticle, so the increment of the LCR in the hair appears disheveled. Thus, we hypothesize that the LCR length relates to the changes in hair coat condition. We examined the length rate of the LCR over the length of the entire hair to determine the relation between the ratio of the LCR and each hair coat condition score. Fig 1D shows the ratio of LCR to the full-length hair for each score. The LCR ratios were 65.6 ± 1.0%, 67.3 ± 3.5%, 73.0 ± 1.3%, and 73.6 ± 1.2% for scores 0, 1, 2, and 3, respectively, and increased in a score-dependent manner. The ratios of LCR in scores 2 and 3 were significantly increased compared with that of score 0.

### Sex differences associated with hair condition changes

**Fig 2:**
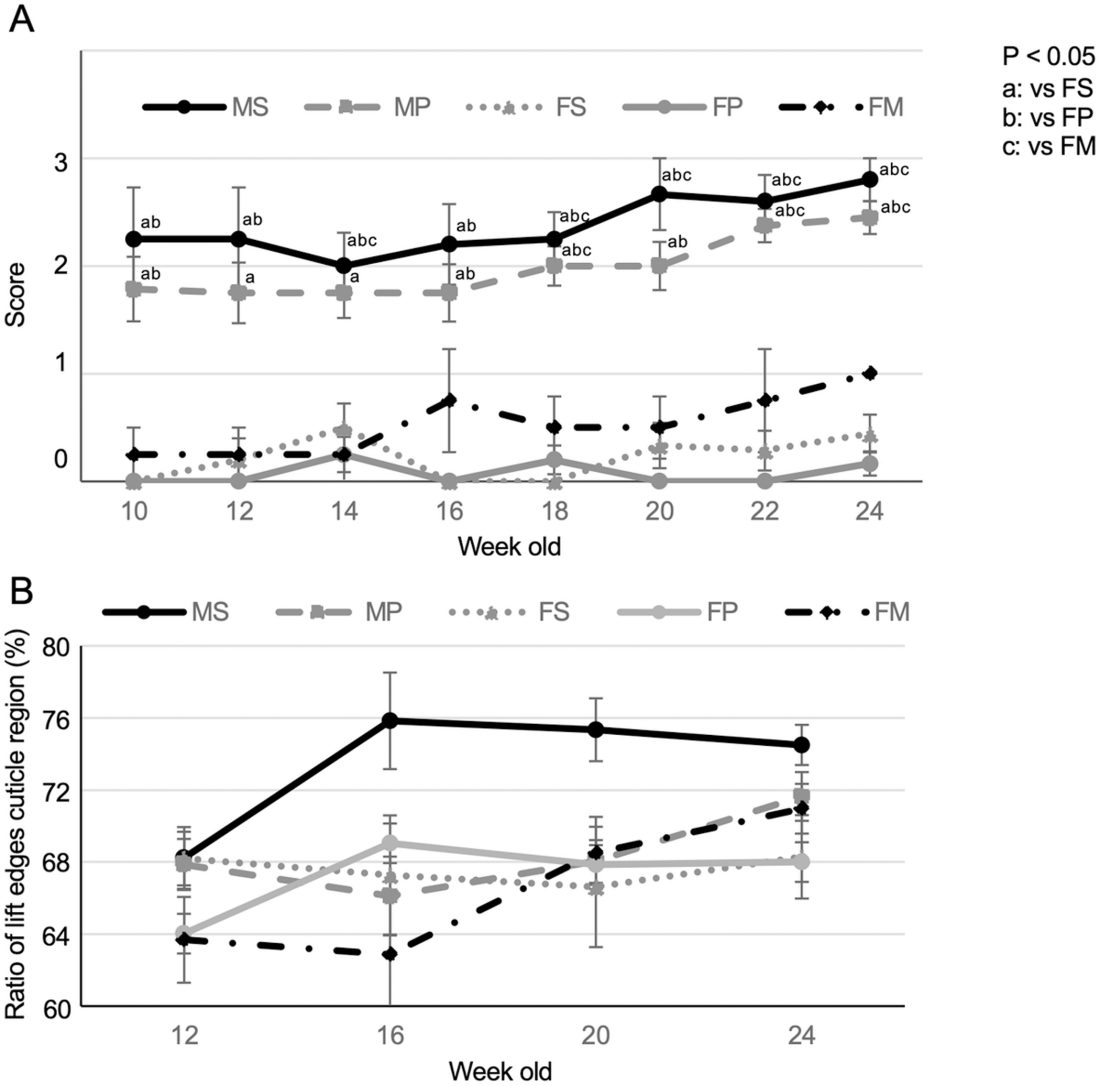
Effect of a change in coat condition score and the ratio of LCR over time for each breeding pattern. (A) Change in the hair coat condition score over time. Different letters indicate significant differences between groups for the same time point. (B) Change in the ratio of LCR over time. MS: male single-breeding; MP: male pair-breeding; FS: female single-breeding; FP: female pair-breeding; FM: female and male breeding. Error bar, SE.

We examined the hair score change based on sex, breeding conditions, and age, to determine the factors associated with hair condition changes. The average scores of single and pair-breeding mice in males increased in a time-dependent manner after 10 weeks, whereas those of females did not change and never exceeded a score of 1 (Fig 2A). The average scores in the males were higher compared with those in females for most ages; the average scores in females never exceeded a score of 1 until 24-week-old. Interestingly, breeding conditions affected the score of males, but not females. The average score for the single-breeding male was always highest during breeding conditions. The average score of the pair-breeding male with a male never exceeded that of the single-breeding male of the same age. The increase in the average score with aging was the same for all males during breeding, including that of the pair-breeding male with a female. Furthermore, the average score of the pair-breeding male with the female was lower than that of the breeding with the males, which was statistically significant at 14, 18, 20, 22, and 24 weeks of age compared with the single-breeding male, and at 18, 22, and 24 weeks of age compared with the pair-breeding male. The pair-breeding male with the female, even at 30 weeks old, showed a score of 1 or 2, but did not exceed a score of 3. These results indicate a factor of changes the score that the individual’s sex and some breeding situations that may affect it, particularly the presence of a female that affects the male score. A high ratio of LCR (74.5 ± 1.1% to 75.8 ± 2.7%) was observed in single-breeding males compared with other breeds at 16, 20, 24-week-olds. There was no difference in the ratio of LCR between the breeding events other than the single-breeding male at 20 weeks of age; however, the ratio of LCR increased in 24-week-old pair-breeding males, breeding with the male (71.6 ± 1.4%) or female (71.0 ± 1.4%) close to the single-breeding male. (Fig 2B). These results indicate that gender is a factor that effects the score and that some breeding situations, such as the presence of a female, can alter the male score.

### Decrement of the hair coat condition score by castration

**Fig 3:**
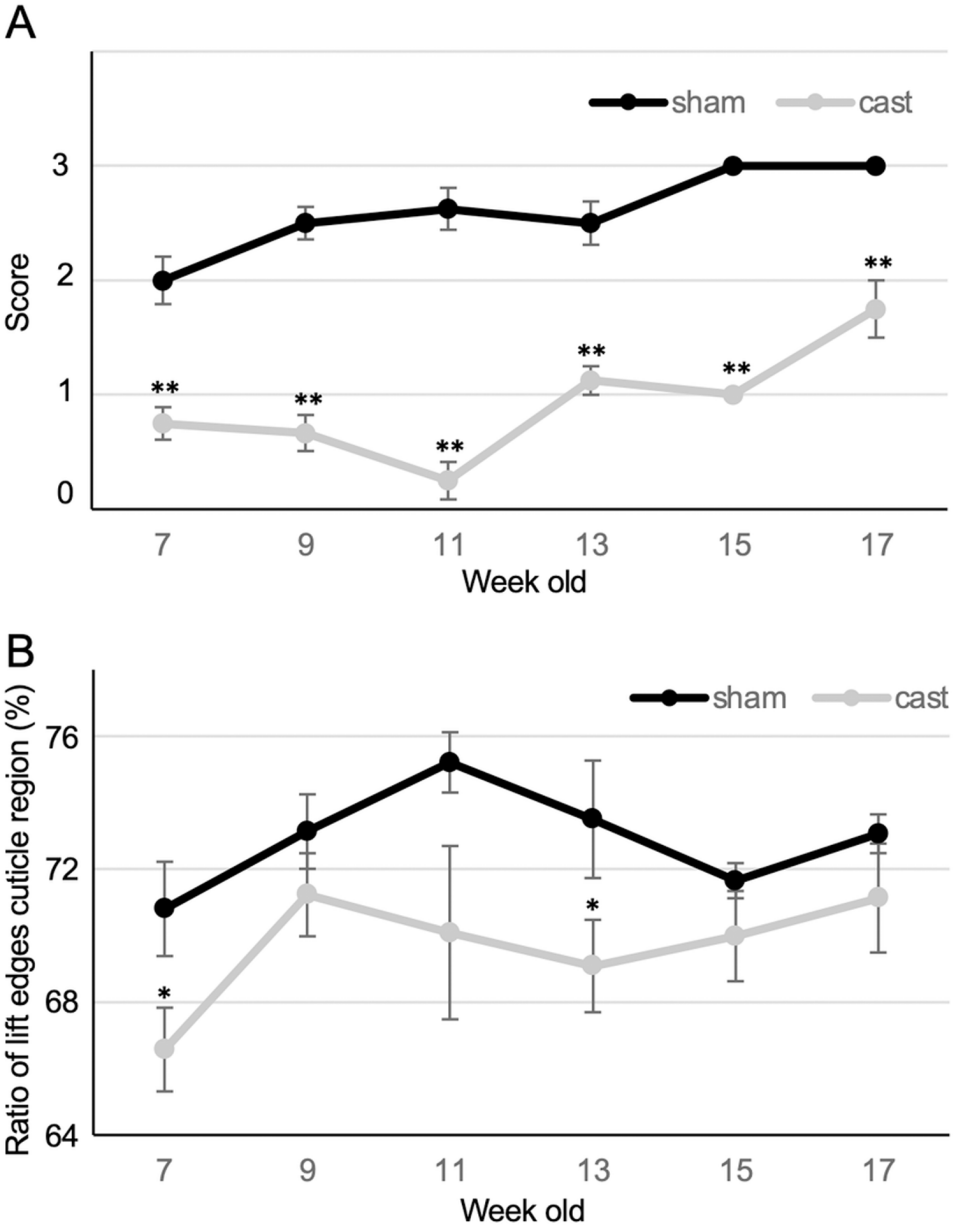
Effect of castration on the hair coat condition score and the ratio of LCR. (A) Change in the hair coat condition score over time. (B) Change in the ratio of LCR over time. Error bar, SE. *P < 0.05, **P < 0.01.

The single-breeding male indicated a score of 2, changing the whole hair coat of the dorsal neck until 10 weeks old, whereas the single-breeding female showed no changes even at 20 weeks of age. Mice are completely sexually mature at 8 weeks. We hypothesized that sexual maturation is a factor for increased hair condition scores in males and we determined whether castration had any effect on the increasing score of males. Mice were castrated at 5 weeks of age to avoid the effect of sexual maturation. The hair coat condition score increased in a time-dependent manner from 2 to 3 between 7 and 17 weeks of age in sham-operated mice. Following castration, the hair coat condition score increased from a score of 1 in 17-week-old mice, but never exceeded a score of 2. The average score in the castrated mice was significantly lower at all ages and the difference was never less than 1 (Fig 3A); moreover, a score of 3 was not observed in castrated males after 12 weeks of treatment. The ratio of LCR in the sham-operated mice and castrated mice changed from 70.8 ± 1.4% to 75.2 ± 0.9% and 66.6 ± 1.3% to 71.2 ± 1.2%, respectively. The ratio of LCR in the castrated mice decreased at 7 and 13 weeks of age and did not exceed those in the sham-operated mice of the same age (Fig 3B). The results indicated that the testes may produce a stimulating factor for hair coat changes in males.

### The hair coat condition shows plasticity

**Fig 4:**
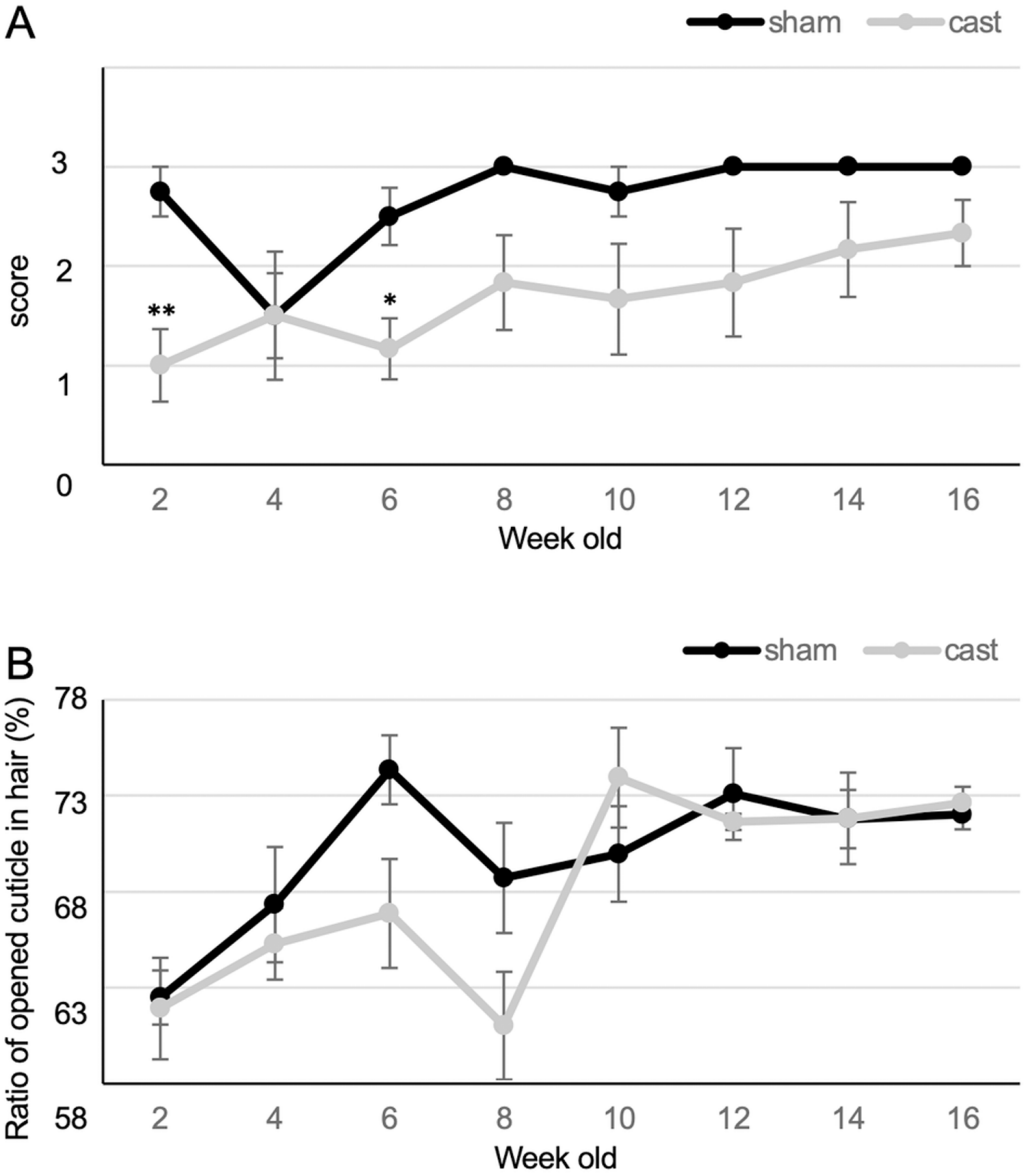
Effect of change in the coat condition score and the ratio of LCR on the coat condition score 3 mice following castration. (A) Change in the coat condition score over time. (B) Change in the ratio of LCR over time. Error bar, SE. *P < 0.05, **P < 0.01.

We performed castration on score 3 mice and observed changing hair coat conditions to determine whether the hair coat condition exhibited plasticity. The hair coat condition score continued to score 3 before the operation in the sham-operated mice; however, the average hair coat condition score decreased to 1 after castration and sustained a lower score average compared with those of the sham-operated mice at 16 weeks following the procedure. Castration significantly decreased the hair coat condition score at 2 and 6 weeks after treatment (Fig 4A). The ratio of LCR in the sham-operated mice and castrated mice increased from 62.5 ± 1.4% to 74.4 ± 1.8% and 61.0 ± 2.8% to 73.9 ± 2.6%, respectively (Fig 4B). The results indicate that the hair coat condition was altered with decrease with the hair coat condition score and the ratio of LCR following castration, indicating the potential plasticity of the hair coat condition.

## Discussion

Animal coats have many roles, including protection of the body, individual identification, and an indicator of physical condition. Physical conditions change the coat state, and poor physical conditions result in rough hair coats [9]. Therefore, the coat condition is a good indicator of an animal’s physical state. This study revealed sex differences in the coat condition between males and females in health condition. Moreover, castration suppressed the increase in coat condition score and the ratio of lift-edges cuticle in males. This indicates that a testicular-derived factor may induce changes in the coat condition. In addition, the relation between the coat condition and the ratio of LCR to indicate the cuticle state showed the cuticle to affect the overall hair coat condition. Although the effects of ultraviolet light and chemicals on cuticle conditions have been studied, few have reported direct changes in cuticle conditions when the overall condition of the hair is altered. The results of the present study suggest that the cuticle may be one of the factors that change the condition of the coat. The state of the lift-edges cuticle observed by microscopy deviated between the cuticle layers. The divergence between the cuticle layers is associated with changes in the fatty acids involved in the bonding between the lipids and cuticles on the hair surface [7]. Therefore, we hypothesize that sebaceous gland secretions that are delivered to the surface of the hair may be related to changes in the condition of the coat. In the future, we will examine the components of the hair surface and the state of the sebaceous glands that induce changes in the cuticle, and then clarify the relation between these components and changes in coat condition. Castration decreased the hair coat condition score and the ratio of LCR; it also resulted in plasticity. This suggests that a factor from the testis may be responsible for the change in appearance. Testosterone is one of the factors from the testis that causes systemic effects as a hormone; it influences the growth of hair, melanogenesis, and maintains the sebaceous glands [1,2,10,11]. Thus, testosterone is an important factor for hair coat conditions. The effects on hair coat condition score and the ratio of LCR by castration may result from changes in hair growth and sebaceous gland secretion, as testosterone levels are reduced. Castration decreased the hair coat condition score, but did not sustain a score of 0 or 1 as observed in females, and increased scores were associated with aging. In addition, increased coat condition score and the ratio of LCR were observed 8 weeks following castration. The results indicate that hair coat condition changes are affected not only by a testicular-derived factor, but also by another factor. The presence of another mouse in the same cage also affected the hair coat condition score for males, which was even more significant compared to the presence of a female in the same cage. The presence of another mouse in the same cage decreased the hair coat condition score in males; this effect was more pronounced during breeding with females, but male pair-breeding also suppressed hair condition. Male mice have aggressive against other male including same litter and male aggressive behaviors are induced via testosterone [12,13]. Fighting caused immediate change for hair coat condition and might give damage for hair structures. On the other hand, previous studies have reported that testosterone does not necessarily increase aggressive behavior in group-housed male mice [13]. Therefore, in this study, it is possible that co-breeding suppressed male aggression by testosterone. Additionally, co-breeding may have encouraged mutual grooming, which could have positively affected hair coat condition, including the integrity of the hair cuticle. The hair coat condition score in females was not affected by breeding with another male or female mouse. The presence of male mouse-induced estrus in multiple breeding females synchronized by volatile male pheromones is known as the Whitten effect [14]. Other male effects on females, the Bruce effect and Vandenbergh effects, have been reported. They influence females through male urinary 17β-estradiol [15]. The effect of a female on the males was associated with increased cortisol levels in the blood in estrus female chimpanzees [16]. Further studies are needed to determine whether there is an effect of grooming or non-contact-like volatile substances on coat conditions.

## Conclusions

Our study suggests that hair coat condition may be affected by hair cuticle condition, with the lift-edged cuticle inducing changes in the appearance of the whole hair coat. In addition, hair coat condition in males may be altered by a factor in the testis and the presence of another mouse, which was more effective for females compared with males. The hair coat condition of animals and human is a factor in establishing an impression. This study indicates that the visual impression may be altered by the biochemical surrounding and the presence of others.

## Supporting information

S1 Data Original data and statistical analyses data for all relevant figures. (XLSX)

## Abbreviations

LCR: lift edges cuticle region
JF1/Ms: Japanese Fancy mouse 1
MS: male single-breeding
MP: male pair-breeding
FS: female single-breeding
FP: female pair-breeding
FM: female and male breeding
